# Laparoscopic pectopexy: the learning curve and comparison with laparoscopic sacrocolpopexy

**DOI:** 10.1007/s00192-021-04934-4

**Published:** 2021-08-18

**Authors:** Fei Chi Chuang, Yu Min Chou, Ling Ying Wu, Tsai Hwa Yang, Wen Hsin Chen, Kuan Hui Huang

**Affiliations:** Department of Obstetrics and Gynecology, Kaohsiung Chang Gung Memorial Hospital and Chang Gung University College of Medicine, No. 123, Dapi Road, Niaosong District, Kaohsiung City, 833401 Taiwan

**Keywords:** Laparoscopy, Learning curve, Pectopexy, Pelvic organ prolapse, Sacrocolpopexy

## Abstract

**Introduction and hypothesis:**

In addition to laparoscopic sacrocolpopexy (LS), laparoscopic pectopexy (LP) is a novel surgical method for correcting apical prolapse. The descended cervix or vaginal vault is suspended with a synthetic mesh by fixing the bilateral mesh ends to the pectineal ligaments. This study was aimed at developing a learning curve for LP and to compare it with results with LS.

**Methods:**

We started laparoscopic/robotic pectopexy in our department in August 2019. This retrospective study included the initial 18 consecutive women with apical prolapse receiving LP and another group undergoing LS (21 cases) performed by the same surgeon. The medical and video records were reviewed.

**Results:**

The age was older in the LP group than in the LS group (65.2 vs 53.1 years). The operation time of LP group was significantly shorter than that of the LS group (182.9 ± 27.2 vs 256.2 ± 45.5 min, *p* < 0.001). The turning point of the LP learning curve was observed at the 12th case. No major complications such as bladder, ureteral, bowel injury or uncontrolled bleeding occurred in either group. Postoperative low back pain and defecation symptoms occurred exclusively in the LS group. During the follow-up period (mean 7.2 months in LP, 16.2 months in LS), none of the cases had recurrent apical prolapse.

**Conclusions:**

Laparoscopic pectopexy is a feasible surgical method for apical prolapse, with a shorter operation time and less postoperative discomfort than LS. LP may overcome the steep learning curve of LS because the surgical field of LP is limited to the anterior pelvis and avoids encountering the critical organs.

## Introduction

Pelvic organ prolapse (POP) is a prevalent disease in aging societies. The cumulative lifetime risk of POP surgery at the age of 80 years has been reported to be 12.6% [1]. In pelvic reconstruction surgery, apical support is an important factor for a successful outcome. When performing anterior colporrhaphy without apical suspension, the reoperation rate for recurrent prolapse significantly increases [2]. Apical suspension can be performed transabdominally or transvaginally using native tissue or a synthetic mesh. Sacrocolpopexy results in less anatomical recurrence than native tissue repairs and transvaginal mesh and lower complication rates than transvaginal mesh [3]. However, laparoscopic sacrocolpopexy requires proficiency in laparoscopic skills, resulting in a steep learning curve, and has a longer operating time than native tissue repairs and transvaginal mesh [4]. The pectineal ligament (Cooper’s ligament) was found to consist of stronger and more durable tissue than the sacrospinous ligament and arcus tendineus of the fascia pelvis in a previous study [5]. Banerjee and Noé first introduced laparoscopic pectopexy (LP) using synthetic mesh anchoring on the bilateral pectineal ligaments in 2011 [6]. Further studies showed comparable outcomes in supporting the apical compartment at intermediate follow-up duration compared with laparoscopic sacrocolpopexy (LS) [7, 8]. Advantages of LP compared with LS are shorter operation time and lower complication rate [7].

Our aim was to determine the learning curve for LP and to evaluate the operation time of LP compared with LS. We also reported our experience with modifications to facilitate the procedure, as well as peri- and postoperative results.

## Materials and methods

We started laparoscopic/robotic pectopexy in our department in August 2019. Before this, laparoscopic/robotic sacrocolpopexy was the major procedure for dominant apical prolapse in our clinical practice. This retrospective study included the initial 18 consecutive women with apical prolapse receiving LP and another group undergoing LS (21 cases) performed by the same surgeon (FCC). According to a study reported by Akladios et al., the operation time of laparoscopic sacrocolpopexy was significantly reduced after 18–24 procedures [4]. Thus, we collected the cases after the 25th LS performed by the same surgeon. A total of 21 women undergoing sacrocolpopexy were enrolled. Patients undergoing concomitant hysterectomy were excluded to reduce the difference in surgical methods. The electronic medical records and video records were reviewed, including patients’ characteristics, operation details, and preoperative Pelvic Organ Prolapse-Quantification (POP-Q) stages. The preoperative POP-Q stage was measured under general anesthesia. The operation time and amount of blood loss were retrieved from the operation records. The operation time was calculated by subtracting the “end of surgery” from the “start of surgery” obtained from the operation record. The operation time included all concomitant procedures (laparoscopy, colporrhaphy, anti-incontinence surgery, and cystoscopy). Perioperative details, including hospital stays, indwelling catheter duration, and pain score evaluated by the Numeric Rating Scale, were also obtained. After discharge from the hospital, follow-up was scheduled at the outpatient clinic at 1 week, 6 weeks, 3 months, and 12 months, and then annually thereafter. Postoperative discomfort including low back pain, low abdominal pain, defecation symptoms, dyspareunia and buttock pain, and complications were documented according to medical records. Institutional Review Board approval was obtained from the Kaohsiung Chang Gung Memorial Hospital (IRB no. 202100177B0).

### Surgical procedures

**Fig. 1:**
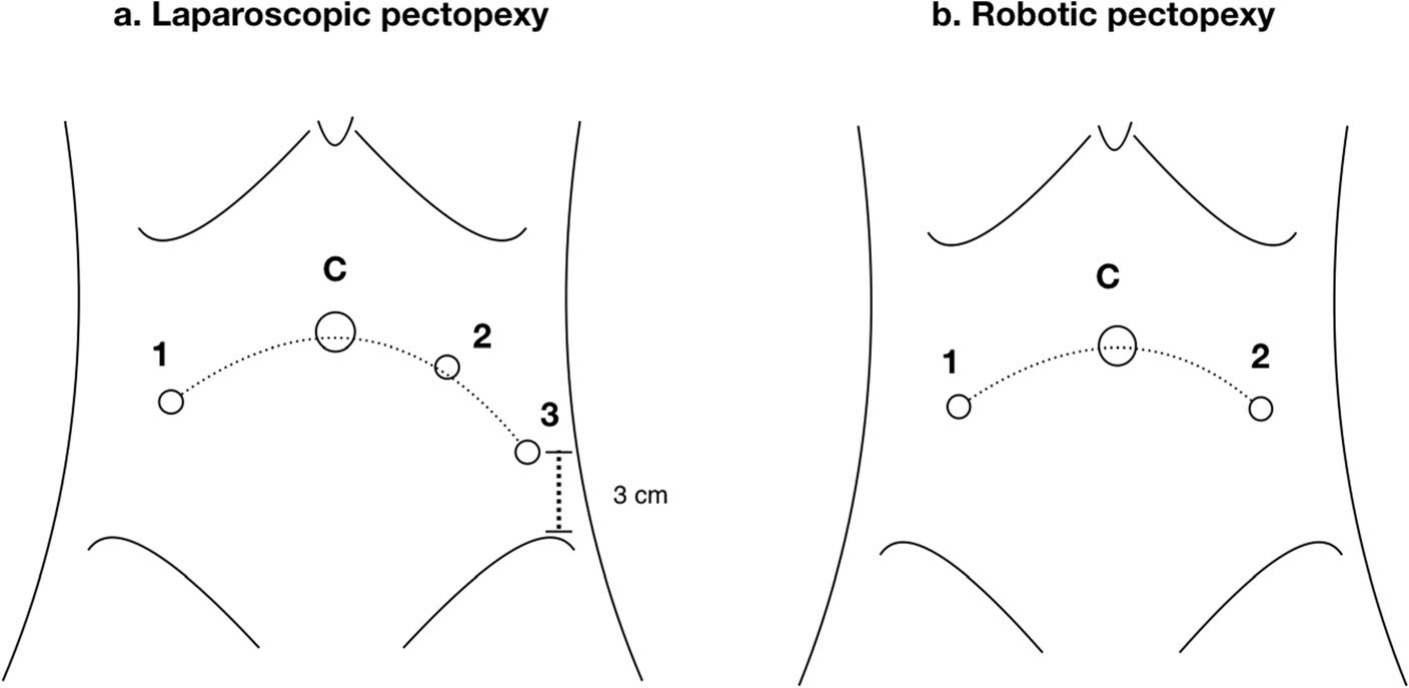
The design of the trocar sites. **a** In laparoscopy, the surgeon used trocars *2* and *3* and the assistant used trocar *1*. *C* camera trocar over the umbilicus. **b** In robotic surgery (Da Vinci Si system), no assistant port was needed. The numbers *1* and *2* represent arms 1 and 3 respectively. *C* camera trocar

All operations were performed using laparoscopy or robotic-assisted systems. First, we used the Veress needle technique for insufflation via the umbilicus to generate pneumoperitoneum to an intra-abdominal pressure of 15 mmHg. The first trocar (10-mm trocar for laparoscopy, 12-mm for the robotic Si system) was inserted through the umbilicus for an endoscopic camera. Three additional 5-mm trocars for laparoscopy, and two 8-mm trocars for robotic surgery, were inserted under direct visualization over the lower abdomen (Fig. 1a). In robotic pectopexy, we used three arms to perform the procedure without any assistant port (Fig. 1b). A uterine manipulator had been placed or a ring forceps holding one piece of a folded 4- × 4-inch gauze had been inserted into the vagina during a previous hysterectomy.

#### Laparoscopic/robotic pectopexy

**Fig. 2:**
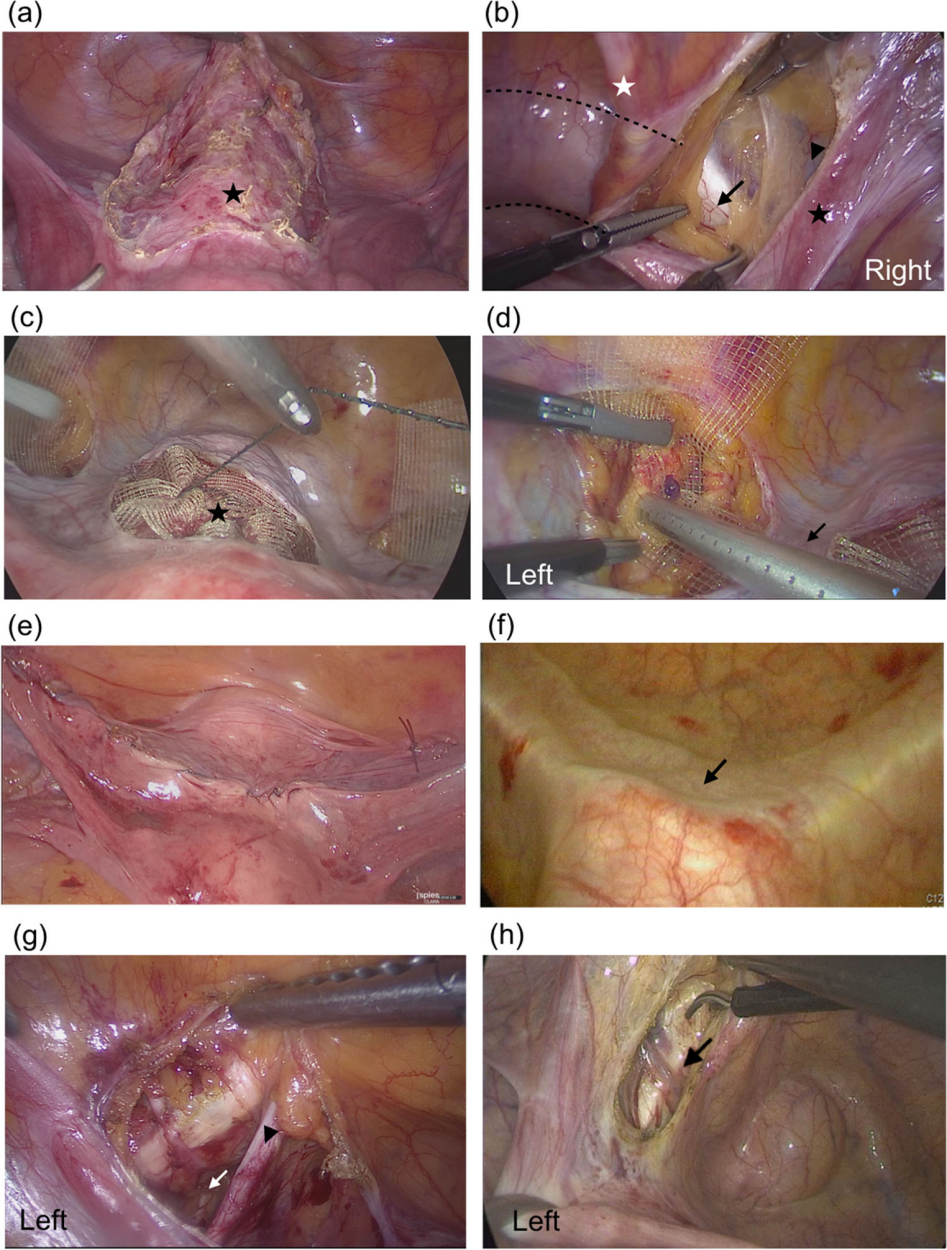
Steps of pectopexy. **a** The peritoneum was opened to dissect the bladder and expose the cervix (*star*). **b** The peritoneum was opened along the pubic bone (*dotted line*) between the right round ligament (*black star*) and the right medial umbilical ligament (*white star*) to expose the pectineal ligament (*arrow*). The external iliac vessels lie on the superolateral part of the pectineal ligament (*arrowhead*). **c** The middle part of the mesh is fixed on the exposed uterine cervix (*star*). **d** The mesh end is anchored to the left pectineal ligament by AbsorbaTack™. The retroperitoneal tunnel from the cervix to the left pectineal ligament (*arrow*). **e** Reperitonization after mesh fixation. **f** Intraoperative cystoscopy. The surface indentation of the pectopexy mesh over the bladder dome (*arrow*). **g** The obturator neurovascular bundle (*white arrow*) is at the inferolateral part of the left pectineal ligament. *Arrowhead*: left medial umbilical ligament. **h** The pubic vein lies on the left pectineal ligament (*arrow*)

The anterior peritoneum of the uterus was opened, and the bladder was dissected to expose the cervix in preparation for mesh fixation (Fig. 2a). In patients with a previous hysterectomy, the peritoneum of the vaginal vault was opened from the apex, and the surrounding soft tissue over the apex was dissected anteriorly and posteriorly. We then opened the peritoneum along the pubic bone between the right round ligament and the right medial umbilical ligament to expose the pectineal ligament. The right pectineal ligament was prepared just medial to the external iliac vessels and was dissected anteriorly for about 3 cm in length (Fig. 2b). The left pectineal ligament was prepared as described above. In the three-arm robotic surgery, we created bilateral retroperitoneal tunnels from the cervix/vaginal vault to the pectineal ligaments, instead of opening the entire peritoneum along the round ligaments. These tunnels could minimize the mesh movement during mesh fixation without assistance. The DynaMesh®-PRS 3 × 23 cm (FEG Textiltechnik mbH, Aachen, Germany) was inserted into the peritoneal cavity. We used 1–O V-Loc 180 (Covidien, Mansfield, MA, USA) to fix the mesh onto the anterior cervix or vaginal vault (Fig. 2c). The uterus or vaginal vault was elevated to the natural position without excessive tension by the manipulator. The mesh ends were anchored to the bilateral pectineal ligaments with two interrupted 2–O Ethibond sutures (Ethicon, Somerville, NJ, USA) or AbsorbaTack™ (Covidien) using 2–3 tacks on each side for mesh fixation of the pectineal ligament (Fig. 2d). The instrument of the AbsorbaTack™ was inserted via the contralateral trocar to apply vertical pressure on the pectineal ligament to yield the appropriate fixation of the tack.

#### Laparoscopic/robotic sacrocolpopexy

The serosa of the uterine cervix or vaginal vault was opened anteriorly and posteriorly down to the vagina. To expose the anterior longitudinal ligament, the peritoneal layer over the sacral promontory was incised, and the overlying adipose tissue was dissected carefully to avoid median sacral vessel injury. A retroperitoneal tunnel was created over the right pelvic side wall to connect the uterine cervix or vaginal vault. The Y-shaped mesh was sutured to the anterior and posterior cervix and vagina with 1–O V-Loc™. The upper end of the Y-shaped mesh was fixed with two or three 2–O Ethibond sutures to the anterior longitudinal ligament [9].

After mesh fixation, the peritoneum was closed continuously (Fig. 2e). Anterior and posterior colporrhaphy was performed if the cystocele or rectocele was higher than POP-Q stage 2 after LP or LS. Anti-incontinence surgery with a mid-urethral sling was performed in patients with stress urinary incontinence (SUI). In patients diagnosed with occult SUI, we counseled them about the risk of postoperative SUI, and the anti-incontinence surgeries were performed at the patients’ discretion. Cystoscopy was performed at the end of the surgery in all cases to check the integrity of the bladder and ureteral urine jets (Fig. 2f).

### Statistical analysis

Categorical variables were expressed as counts and percentages. Continuous variables were expressed as means and standard deviations. To compare the characteristics between groups, the Chi-squared test was used for categorical variables, and paired Student’s *t* tests or Mann–Whitney *U* tests were used for continuous variables. Differences were considered statistically significant when the *p* value was <0.05.

We used cumulative sum (CUSUM) analysis to establish the learning curve of LP. The predefined level was the average operation time. We counted the difference between every operation time and the average operation time and depicted the curve using accumulative differences. If the surgery was longer or shorter than the average operation time, the curve would rise or fall accordingly. The turning point was where the positive slope became negative. All data were analyzed using SPSS (Version 22.0 IBM Corp, Armonk, NY, USA) for Windows. The learning curve was illustrated using Microsoft Excel (2016 for Mac).

## Results

The patients’ demographic data are listed in Table 1. Patients who received pectopexy were older. The pectopexy group included more women who had undergone vaginal deliveries, more women with menopausal status, and more women with hypertension, which was consistent with the older age. There was no difference in the preoperative POP-Q stage between the two groups. Both groups had the majority of patients with stage 3 cystocele and stage 3 apical prolapse.

**Table 1:** Patients’ demographics

	Pectopexy (*n* = 18)	Sacrocolpopexy (*n* = 21)	*p* value
Age (years)	65.2 ± 8.8	53.1 ± 11.5	0.001*
BMI (kg/m^2^)	24.9 ± 2.9	24.3 ± 3.3	0.568
Parity	2.7 ± 1.2	2.2 ± 0.9	0.14
Vaginal delivery	2.7 ± 1.2	2.1 ± 0.8	0.069
Cesarean delivery	0.06 ± 0.24	0.06 ± 0.24	0.835
Diabetes mellitus	1 (5.6)	2 (9.5)	1.0
Hypertension	9 (50)	4 (19)	0.041*
Menopause	17 (94.4)	13 (61.9)	0.023*
Pelvic surgery history	4 (22.2)	3 (14.3)	0.682
Prior POP surgery	1 (5.6)	1 (4.8)	1.0
Myomectomy	1 (5.6)	1 (4.8)	1.0
Hysterectomy	2 (11.1)	1 (4.8)	0.586
Preoperative POP-Q stage			
Cystocele stage 2/3/4	2/13/3 (11.1/72.2/16.7)	4/16/1 (19.0/76.2/4.8)	0.442
Apical prolapse stage 2/3/4	1/12/5 (5.6/66.7/27.8)	3/16/2 (14.3/76.2/9.5)	0.256
Rectocele stage 2/3/4	10/7/1 (55.6/38.9/5.6)	8/11/1 (38.1/52.4/4.8)	0.703

Mean ± standard deviation, *n* (%)

*BMI* body mass index, *POP* pelvic organ prolapse, *POP-Q* Pelvic Organ Prolapse Quantification

*p value <0.05

Table 2 showed the surgical details of LP and LS. The operation time of LP group was significantly shorter than that of the LS group (182.9 ± 27.2 vs 256.2 ± 45.5 min, *p* < 0.001). More adnexal surgeries were performed in the LS group, but not statistically significantly. The concomitant surgeries in the LS group included excision of a paratubal cyst, salpingectomy, and ovarian cystectomy. All these procedures were simple and took less than 15 min to perform. The mean operation time of robotic-assisted pectopexy was 184.5 min, but this was not significantly different from conventional laparoscopic pectopexy (178.5 min).

**Table 2:** Surgical and perioperative details

	Pectopexy (*n* = 18)	Sacrocolpopexy (*n* = 21)	*p* value
Laparoscopy	8 (61.5)	16 (76.2)	0.51
Robotic-assisted	6 (33.3)	5 (23.8)	
Concomitant procedure			
Colporrhaphy	14 (77.8)	14 (66.7)	0.442
MUS	3 (16.7)	4 (19.0)	1.0
Adnexal surgery	1 (5.6)	6 (28.6)	0.098
Adhesiolysis	1 (5.6)	1 (4.8)	1.0
Rectal suspension	1 (5.6)	0	0.462
Blood loss (ml)	33.6 ± 39.3	23.1 ± 14.8	1.0
OP time (min)	182.9 ± 27.2	256.2 ± 45.5	<0.001*
Hospital stays (days)	2.8 ± 0.9	3.1 ± 0.9	0.335
Indwelling Foley catheter duration (days)	1.3 ± 0.5	1.4 ± 0.5	0.587
Numeric rating scale			
Operation day	3.2 ± 1.5	2.8 ± 0.5	0.257
Postoperative day 1	2.6 ± 0.8	2.1 ± 0.8	0.106
Postoperative day 2	2.1 ± 0.6	1.6 ± 0.9	0.157

Mean ± standard deviation, *n* (%)

*MUS* mid-urethral sling, *NRS* Numeric Rating Scale

*p value <0.05

In the pectopexy group, 10 patients had AbsorbaTack™ for mesh fixation on the pectineal ligaments, and the remaining patients had Ethibond sutures. During the mean 7.2-month follow-up (range 1–16.4 months), the C points of all pectopexy patients showed excellent results. The lowest C point was −5, which was no more than −1/2 of the total vaginal length. In the sacrocolpopexy group, none of the patients had apical recurrent prolapse at the mean 16.2-month follow-up.

**Fig. 3:**
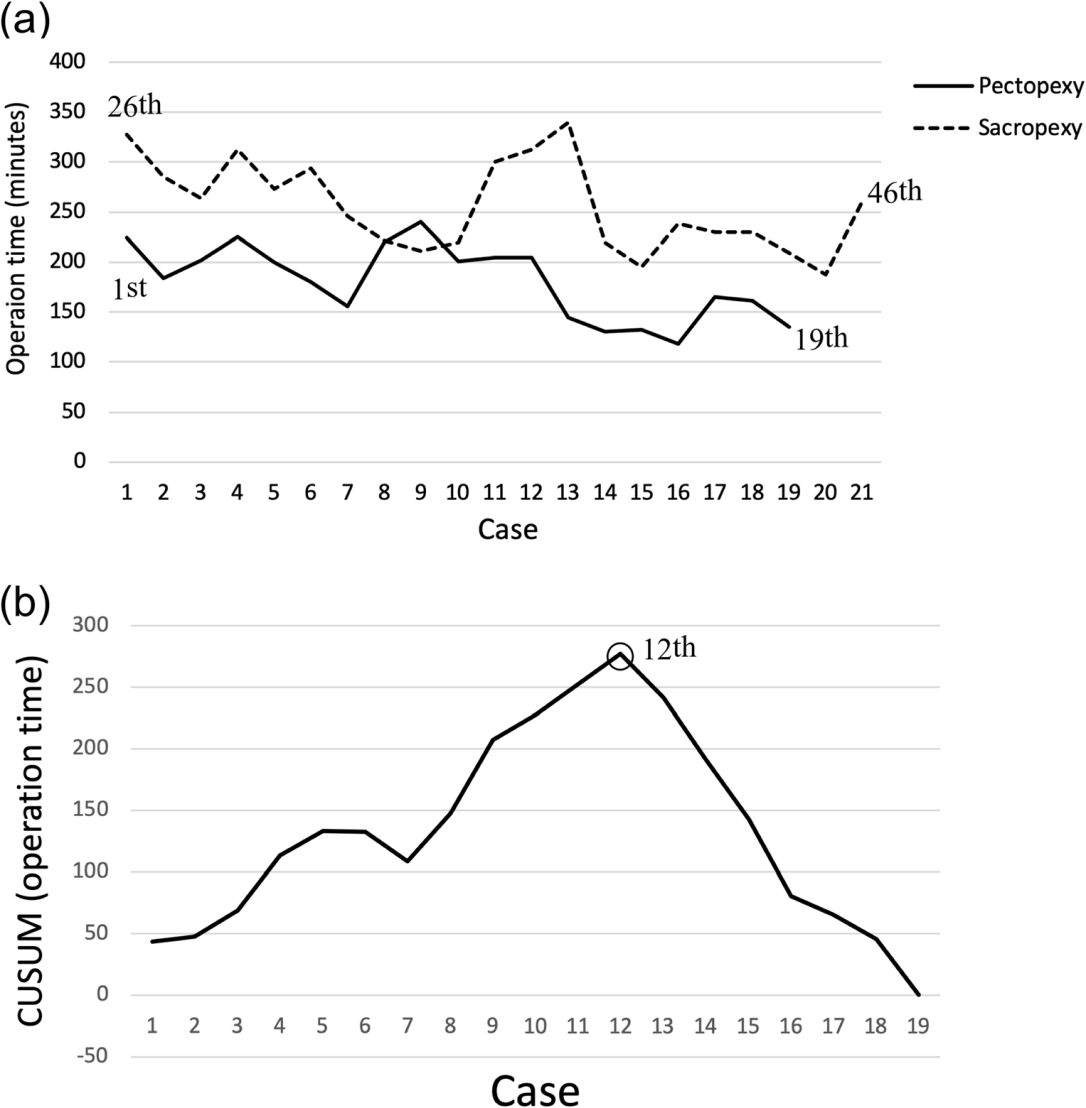
Distribution of the operation time. **a** Operation time of laparoscopic pectopexy and laparoscopic sacrocolpopexy. **b** Learning curve of laparoscopic pectopexy. The turning point of the learning curve was the 12th case

The operation time comparing the two groups is shown in Fig. 3a. The operation time of pectopexy showed a less fluctuating pattern than sacrocolpopexy, and it had been shorter since the beginning. The operation time could be further reduced as the surgeon obtained more experience. The learning curve of LP clearly showed that the turning point was the 12th case (Fig. 3b).

Postoperative follow-up results are listed in Table 3. Low back pain and defecation symptoms including constipation and dyschezia occurred exclusively in the sacrocolpopexy group. One patient with failed conservative treatment for low back pain had reoperation after 4 months to excise 2 cm of the mesh from the sacral promontory. Her pain improved immediately after surgery. The other three patients improved with medical treatment. Six patients developed postoperative SUI after pectopexy. Four of them had occult SUI detected in a preoperative urodynamic study and did not undergo concomitant anti-incontinence surgery. There were no major complications, including bladder, ureteral, bowel injury or uncontrolled bleeding, in either group.

**Table 3:** Follow-up results

	Pectopexy (*n* = 18)	Sacrocolpopexy (*n* = 21)	*p* value
Follow-up duration (months)	7.2 (1–16.4)	16.2 (1–41.7)	
Low back pain/soreness	0	4 (19.0)	0.11
Low abdominal pain/soreness	2 (11.1)	4 (19.0)	0.667
Defecation symptoms^a^	0	4 (19.0)	0.11
Dyspareunia	0	2 (9.5)	0.49
Postoperative SUI	6 (33.3)	2 (9.5)	0.112
De novo urgency	0	2 (9.5)	0.49
Buttock pain	0	1 (4.8)	1.0
Mesh exposure	0	0	n/a
Urinary tract injury	0	0	n/a
Bowel injury	0	0	n/a

Mean (range), *n* (%)

*n/a* not applicable *SUI* stress urinary incontinence

*p value <0.05

^a^Defecation symptoms in sacrocolpopexy include constipation (*n* = 3) and dyschezia (*n* = 1)

## Discussion

Laparoscopic sacrocolpopexy has been shown to be a durable procedure for apical prolapse, which has a less negative impact on sexual function [10, 11]. However, the steep learning curve of LS is an obstacle for a novice. Laparoscopic pectopexy was introduced in 2011 and showed that the procedure offers a feasible, safe, and easier to perform alternative for apical prolapse surgery [6]. A prospective, randomized, comparative clinical trial of standard LS (*n* = 41) with the new LP (*n* = 44) also found that LP offers clear practical advantages and possesses comparable recurrence rates (LS vs LP, 9.8% vs 2.3%) [8]. Our study also demonstrated that the operation time of LP was 73.3 min shorter than that of LS, which was performed by a skilled surgeon. Compared with other studies [7, 12], our operation time of LP was longer. This point may be due to the fact that we counted the operation time to include all the procedures, such as colporrhaphy, anti-incontinence surgery, and cystoscopy.

Postoperative follow-up results revealed that low back pain and defecation symptoms occurred exclusively in the LS group. The reported incidence of de novo low back pain after sacrocolpopexy is 18%, which is similar to our findings [13]. Most patients can be treated conservatively with medication and/or physiotherapy. Patients in whom conservative treatment fails should raise the concern of spondylodiscitis [14]. Sacrocolpopexy reduces the posterior pelvic space and may cause hypogastric nerve injury that results in defecation problems, whereas constipation is the most common symptom. Our defecation symptoms rate was similar to that of a previous study [8].

A higher incidence of postoperative SUI (*n* = 6, 33.3%) was observed in our pectopexy group. Four of them were diagnosed as having occult SUI according to the preoperative urodynamic study. Occult SUI is a known risk factor for postoperative SUI in the case of POP surgery without a concomitant anti-incontinence procedure [9]. None of our patients reported de novo urgency or unusual lower urinary tract symptoms.

The learning curve of pectopexy, based on operation time, showed the turning point at the 12th case. The reason why fewer cases were needed in pectopexy to achieve the turning point than sacrocolpopexy may be due to anatomical differences in the procedures. The adjacent important landmarks during dissection of the pectineal ligament are the external iliac vessels and obturator nerve (Fig. 2g). A cadaver study showed that the mean distance from the midpoint of the pectineal ligament to the external iliac vein was 1.04–1.25 cm and to the obturator canal was 3.12–3.57 cm [15]. The obturator nerve passes below the pectineal ligament and is relatively distant from the dissection plane. However, caution is still needed during dissection, especially during cauterization. The external iliac vessels are close to the operative field, but are easy to identify by their obvious color and pulsation before incising the peritoneum. A vessel that may be encountered during pectineal ligament preparation is the pubic vein, also called the corona mortis (Fig. 2h), which is the anastomosis of the external iliac vein and obturator vein and lies on the pectineal ligament [15]. It can simply be cauterized if this vessel is impeding the mesh fixation.

From the experience of exploring the LP procedure, we found that using tacks on the pectineal ligament is a feasible alternative to suture. The one-shot step of AbsorbaTack™ is quite time-saving compared with the complicated process of laparoscopic suture, especially for a novice. The pectineal ligament is thicker than the anterior longitudinal ligament. The mesh is fixed to the pectineal ligament between the pubic tubercle and the external iliac vessels and the ligament is 4–5 mm thick in this area [16, 17]. It is also the region where the mesh was fixed in pectopexy. The vertical length of AbsorbaTack™ is 4.1 mm. Taking the DynaMesh thickness (0.4 mm) into account, the pectineal ligament is thick enough to drive in the AbsorbaTack™. A previous study showed that the mean depth of needle penetration over the sacral promontory during laparoscopic sacrocolpopexy was 3.96 mm [18]. The anterior longitudinal ligament thickness over the promontory is only 1.9 mm where penetration of the periosteum is more likely to happen [19]. For the reason above, spondylodiscitis after sacrocolpopexy has been reported [14, 20, 21]. It is a rare but devastating complication for patients. Further operation to excise the sutures or for debridement of the bone tissue may be necessary. As mentioned above, AbsorbaTack™ is a reliable alternative fixation tool and has a low risk of osteitis owing to the anatomy of the pectineal ligament.

Another concern for the utilization of absorbable suture is durability. In our LP cases, we used DynaMesh, consisting of polyvinylidene fluoride, which requires 21 days for tissue integration [22]. AbsorbaTack™ started significant absorption after 90 days and was completely absorbed in 12 months. Its delayed absorbable property provides sufficient duration for tissue integration into the mesh pores [23]. None of our patients had recurrent apical prolapse during the follow-up period. From our perspective, AbsorbaTack™ seems to be a reliable alternative fixation tool in LP procedure.

Pectopexy also offers advantages over sacrocolpopexy in obese patients. In sacrocolpopexy, there are several important structures including the right ureter, hypogastric nerves, middle sacral vessels, and left common iliac vein over the sacral promontory. Retroperitoneal dissection for anterior longitudinal ligament preparation and bowel handling is challenging in obese patients because of difficulties identifying major landmarks [19, 24]. Obesity also increases the surgical difficulty because of the limited surgical field in balancing sufficient abdominal pressure and adequate ventilation [25]. In contrast to sacrocolpopexy, pectopexy limits the surgical fields in the anterior pelvic space and is less influenced by obesity.

Our study had limitations, including its retrospective design, small number of cases, relatively short follow-up period, and the learning curve of pectopexy was developed in a surgeon who was already experienced in LS. However, this is to our knowledge the first study investigating the learning curve and some surgical modifications of LP. Our results showed that LP is a reliable procedure without a steep learning curve. We also provided an alternative and convincing fixation technique with autosuture absorbable tack, which may further reduce operation time for surgeons not so familiar in conventional laparoscopic suturing. Creating a retroperitoneal tunnel to connect the bilateral pectineal ligaments to the cervix or the vaginal apex could minimize mesh movement when fixing the mesh ends to the pectineal ligaments. This made three-arm robotic pectopexy feasible without an assistant port. LP is a novel and promising technique. However, long-term and large-scale studies are still needed to verify the efficacy of the surgical modifications introduced in our study.
